# Novel Insights into the Role of Probiotics in Respiratory Infections, Allergies, Cancer, and Neurological Abnormalities

**DOI:** 10.3390/diseases9030060

**Published:** 2021-09-02

**Authors:** Igori Balta, Eugenia Butucel, Valentyn Mohylyuk, Adriana Criste, Daniel Severus Dezmirean, Lavinia Stef, Ioan Pet, Nicolae Corcionivoschi

**Affiliations:** 1 Bacteriology Branch, Veterinary Sciences Division, Agri-Food and Biosciences Institute, Belfast BT4 3SD, Northern Ireland, UK; igori.balta@gmail.com (I.B.); Butucel.eugenia96@gmail.com (E.B.); 2 Faculty of Animal Science and Biotechnologies, University of Agricultural Sciences and Veterinary Medicine, 400372 Cluj-Napoca, Romania; adriana.criste@usamvcluj.ro (A.C.); ddezmirean@usamvcluj.ro (D.S.D.); 3 Faculty of Bioengineering of Animal Resources, Banat University of Animal Sciences and Veterinary Medicine—King Michael I of Romania, 300645 Timisoara, Romania; Lavi_stef@animalsci-tm.ro; 4 School of Pharmacy, Queen’s University Belfast, Belfast BT7 1NN, Northern Ireland, UK; v.mohylyuk@qub.ac.uk

**Keywords:** probiotic, anti-viral, anticancer, psychobiotics, mechanisms

## Abstract

In recent years, probiotics have attracted public attention and transformed the social perception of microorganisms, convening a beneficial role/state on human health. With aging, the immune system, body physiology, and intestinal microbiota tend to change unfavorably, resulting in many chronic conditions. The immune-mediated disorders can be linked to intestinal dysbiosis, consequently leading to immune dysfunctions and a cluster of conditions such as asthma, autoimmune diseases, eczema, and various allergies. Probiotic bacteria such as *Lactobacillus* and *Bifidobacterium* species are considered probiotic species that have a great immunomodulatory and anti-allergic effect. Moreover, recent scientific and clinical data illustrate that probiotics can regulate the immune system, exert anti-viral and anti-tumoral activity, and shields the host against oxidative stress. Additionally, microbiota programming by probiotic bacteria can reduce and prevent the symptoms of respiratory infections and ameliorate the neurological status in humans. This review describes the most recent clinical findings, including safe probiotic therapies aiming to medicate respiratory infections, allergies, cancer, and neurological disorders due to their physiological interconnection. Subsequently, we will describe the major biological mechanism by which probiotic bacteriotherapy expresses its anti-viral, anti-allergic, anticancer, and neuro-stimulatory effects.

## 1. Introduction

Historically the concept “probiotic” was initially proposed by the Nobel laureate and a biologist of Moldavian and Jewish origin, namely Ilya Ilyich Mechnikov (Élie Metchnikoff) [1,2]. In 1908 Élie Metchnikoff disclosed that the consumption of fermented dairy products inoculated by starter cultures of *Lactobacillus bulgaricus* and *Streptococcus thermophilus* appeared to improve intestinal microbiota and was associated with promoting longevity in Bulgarian farmers [3]. Lately, in 1912 bacterial-derived probiotic product lactobacillin was promoted by Metchnikoff as a remedy that improves human health and longevity [4]. Nevertheless, his claims installed the first foundations for the scientific basis on investigating the potential of good bacteria before the real research of probiotics was initiated.

However, the actual term was firstly originally coined only in 1965 by *Lilly and Stillwell*, which denoted probiotics as “the substances produced by microorganisms that promote the growth of other microorganisms” and translated from the Greek language that means “*For life*”. Furthermore, in 1989 *Fuller* re-defined probiotics as the “microorganisms that confer beneficial effects to the host via improving their intestinal microbial balance”. Nowadays, the probiotic status is in concordance with the *World Health Organization* (WHO) and *United Nations* (UN), which validated the official definition of probiotics as “microbes, if administered in adequate amounts exert positive effects on host”.

Many reviews have been published in the last decades discussing the effect of probiotics in counteracting bacterial infections (e.g., *Campylobacter jejuni*, *Salmonella enteritidis*, *Clostridium perfringens*, *Escherichia coli*, *Shigella* spp., *Listeria* spp., etc.), especially from intestinal microbiota level [5,6,7]. From these considerations, probiotic bacteria secrete different inhibitory peptides, organic acids, peroxides, including other small-weigh molecules with exceptional anti-bacterial, anti-viral, anticancer, anti-inflammatory activity [8]. Earlier studies demonstrated probiotics’ ability to improve the gut barrier function, to provide a competitive behavior against pathogenic microbiota by restricting adherence of foodborne bacteria to the gut, and ultimately regulate the host’s immune response through the initiation and expression of specific genes [9]. In light of the preceding, a range of new concepts has been recently evolved by representing expanded bacterial communication between the gastrointestinal tract and brain (microbiota-gut-brain axis) involved in neurological disorders and lungs (gut-lung axis), which is involved in respiratory diseases. [10,11]. Furthermore, the approach involving the application of solo probiotic strains or their blended formulations is a promising candidate to successfully mitigate and prevent several diseases.

## 2. Prophylactic Effects of Probiotics in Respiratory Infections

We live in a world facing approximately 200 species of viral pathogens able to infect humans, and most of them are originated from animals harboring a multiplicity linear expanding rate of 3 or 4 strains per year [12]. Currently, humanity is facing novel viral infectious diseases paving the way to urgently revise or develop new and efficient strategies to counter-attack the peril due to their ability to suffer genetic modifications in a relatively short time [13]. The most common and prevalently infectivity pattern is assigned to the viral respiratory tract infections induced by rhinoviruses, coronaviruses, and influenza that usually cause pulmonary diseases worldwide. In children and infants, pathogen-induced pneumonia leads to increased hospitalizations and deaths, usually causing approximately 120 million cases and almost 1 million deaths per year [14]. In just one year, the appearance of a novel coronavirus strain (COVID-19) has affected more than 2 million humans (20 April 2020) worldwide, advocating for the urgent development of novel anti-viral and antimicrobial strategies [15]. Probiotics are known to alleviate and prevent respiratory infections and are frequently reported in the literature (Table 1).

Probiotics, prebiotics, phytobiotics, and natural antimicrobials, including their metabolites, have received great attention, especially due to the SARS-CoV-2 pandemic, and are continuously tested for their ability to inhibit viruses and prevent their pathogenic impact on the host [24]. In the context of this review, probiotics are abundantly reported to express anti-viral activity through different molecular mechanisms. Besides the molecular mechanisms mainly exhibited by protecting the gastrointestinal tract, probiotics protect different organs, including the lungs. For example, the gut-lung cross-talk hypothesis might be involved, highlighting the probiotic-assisted mitigation of pathogenesis of acute respiratory infections by advancing the immune response to eradicate the pathogens and exhibit anti-inflammatory activity, reducing or preventing tissular damage [25].

### 2.1. Preclinical Effect

Many recent reports have detected the SARS-CoV-2 (COVID-19) RNA in the feces of infected patients, which displayed negative results for their respiratory samples that may indicate the cross-talk between the gut-lung axis [11]. Suggestively, this evidence provides some insights into how probiotic interference may positively alter the gastrointestinal environment and defend the respiratory system. The instability between T-helper 17 (Th17) cells and regulatory T-cells (Tregs) has been associated with numerous illnesses involved in inflammatory or infectious conditions [16,26]. The Treg activity can also benefit the host by substantially diminishing the pathogen-induced damage by shielding the lung tissue and preserving lung functionality. This effect was described in a recent human clinical trial, the administration of a probiotic product (*B. subtilis* and *S. faecium*) was shown to facilitate the promotion of Tregs by suppressing the expression of the Th17 cell/Treg ratio after two weeks of treatment [16]. Moreover, the treatment showed a greatly reduced inflammation by normalizing pro-inflammatory parameters and displayed to benefit the overall clinical symptomatology picture of the individuals infected with pneumonia.

At the moment, government funding is mainly focused on testing or inventing novel drugs, but equally importantly, they have to start funding more probiotic and prebiotic (symbiotic) trials to accelerate the global strategy to flatten the pandemic curve [24]. So far, a recent clinical trial conducted by Italian doctors in the “trenches” within the first Italian battle against the COVID-19 outbreak demonstrated that oral bacteriotherapy administration of a mixed probiotic formulation (*L. acidophilus* DSM 32241, *L. plantarum* DSM 32244, *L. helveticus* DSM 32242, *L. brevis* DSM 27961, *L. paracasei* DSM 32243, *B. lactis* DSM 32246, *B. lactis* DSM 32,247 and *S. thermophilus* DSM 32345) in COVID-19 hospitalized patients reduced the risk of respiratory failure by approximately 8-folds, improved gut symptomology and promoted the disappearance of diarrhea in all the subjects within seven days [17]. The authors specified that bacterial strains included in the formulation can intensify the production of nuclear factor (Nrf-2) and the target gene (HO-1), both molecules bearing an efficient anti-viral activity by reducing the oxidative stress through neutralization of the free radicals. This was the first study providing evidence about the introduction of a probiotic blend in combination with standard care presented to decrease mortality and morbidity in COVID-19 patients [17]. Such evidential facts show the many promising avenues can be offered by probiotics and help combat respiratory diseases, suggesting that the bacteria assisted therapy versus COVID-19 burden could play a significant role in the future and become of great importance [27].

Among the other postulated probiotic mechanisms engaged in host protection or amelioration of viral respiratory diseases, the major roles are reported to: reinforce and protect the mucosal barrier; to stimulate the formation of antimicrobial compounds (e.g., H_2_O_2_, lactic acid, bacteriocins, mucins, coagulation molecules); protect against antioxidative stress; reduce chloride production; intervene in the adhesive and replication processes; regulate adaptive, mucosal (toll-like receptors (TLR), T-lymphocytes, cytokines, chemokines and nuclear factor-κB (NF-κB)) and innate immune responses (immunoglobulins, interleukins and Th1/Th2); to reduce the viral entry into mammalian host cells by trapping or binding to virus particles (virions) [12,16,28,29]. Probiotics with the potential to impact the immune system are also known as immunobiotics, and previously described investigations have proposed to influence respiratory immunity through the amelioration of IFNs production, the ability to increase mucosal and systemic antibodies in lungs, and to raise the levels and performance of antigen-presenting cells (APCs), T-cells, and NK cells [12,30]. The route of how probiotic bacteria regulate the innate immune system occurs via TLR, and other signaling pathways play a valuable role in decreasing inflammatory or oxidative processes [27]. Other interesting features of probiotics involve the regulation of the initiation of host inflammasomes that were already subjected to inflammation due to previous viral infections [31]. The host regulation of the innate immunity can be maintained due to an increase in phagocytic and leukocytic activity (monocytes and polymorphonuclear), plus the expression of receptors that are intercalated with phagocytosis and the bactericidal function of neutrophils [12]. Furthermore, TLR ligands, peptidoglycan, muramyl dipeptide, and lipoteichoic acid, including other components, accentuates the immunomodulatory consequences of probiotic therapy [11]. In other words, the therapeutic application of probiotics can constrain viral infections through the fortification and restoration of the mucosal immune system through short-chain fatty acids (SCFA) production.

Furthermore, it is considered that SCFA might confer protection against airway inflammations and allergies. For example, the ability to enhance the integrity of tight junctions via butyrate production, a primary combustible for colonocytes, might suggest a decrease in SARS-Cov-2 invasiveness [32]. The SARS-CoV-2 infection is known to trigger a “cytokine storm” that degenerates the lung of infected patients [19]. Thus the use of probiotics with redox homeostasis and immunomodulatory potential could potentially contribute to COVID-19 mitigation through the regulation of humoral and cellular immune responses that can lead to inhibition of disease progression [19].

### 2.2. Clinical Effect

Due to the lack of safe and efficient treatments, ongoing studies worldwide focused on whether modification of the gut microbiota, with dietary interventions, can be a promising additional complement to the current inventory of Sars-Cov-2 and other viral treatments. Clinical studies suggested distinct evidence based on probiotic supplementation that may play an intrinsic role in the decline of the coronavirus burden. Several meta-analysis investigations based on numerous clinical trials have recently suggested that the consumption of probiotic strains of *Lacticaseibacillus casei* Shirota (*L. casei*), *Lacticaseibacillus. rhamnosus* GG (*L. rhamnosus*), *Lactiplantibacillus. plantarum* (*L. plantarum*) and *Limosilactobacillus reuteri* (*L. reuteri*), and *Bifidobacterium* (*B. infantis*, *B. longum*, and *B. lactis*) will decrease acute respiratory tract infections, including COVID-19, and were able to reduce antibiotic-associated diarrhea and improve the quality of life index (QOL) of patients in the control and placebo groups [19,33,34]. An interesting meta-analysis study concluded that probiotics, mostly *Lactobacillus* and *Bifidobacterium* spp., were able to decline the occurrence of common acute infections in children and infants and were suggested to remarkably decrease the necessity of antibiotic treatments [35].

Earlier studies have suggested that the daily intake of fermented dairy products containing *L. casei* (LcS-FM), *L. paracasei* N1115, and *L. plantarum* DR7can diminish the risk of acute upper tract infections (cold and influenza-like) by boosting the immunity involving a mechanism that triggers the T-cell-mediated natural immune defense [22,36,37]. Similarly, a randomized, double-blind controlled study previously reported that oral administration of heat-inactivated *L. lactis* JCM 5805 mitigated the prevalence of influenza and common cold symptoms through a mechanism involving upregulation of IFN-related genes expression and plasmacytoid dendritic cell (pDC) activation [20]. Suggestively, daily probiotic (*L. delbrueckii* subsp. *bulgaricus* OLL1073R-1) yogurt administration to weakened immune elder patients from nursing homes alleviated the infection by influenza A virus subtype H3N2 by improving the generation of salivary immunoglobulin A (IgA) levels [21]. A similar anti-infectivity pattern was observed in elderly residents who received yogurt enriched with (*L. paracasei* N1115), which intensified the T-cell-mediated natural immune defense by increasing the production of CD3+ cells in comparison to control patients [22]. Finally, in the light of the COVID framework, another prominent benefit of probiotics (e.g., *L. rhamnosus* GG, *B. subtilis*, *E. faecalis*, etc.) is refers to their potential to notably decrease and prevent the incidence of ventilator-related pneumonia in patients who were subjected to mechanical ventilation [15,23,24]. In conclusion, a plethora of in vitro and in vivo studies to date revealed plausible examples of immunobiotics (e.g., *L. plantarum* MPL16 and *L. rhamnosus* CRL1505) with anti-viral potential that could facilitate furtherly efficient weaponry to combat COVID- 19 but needs further investigations involving human trials [30].

## 3. The Effect of Probiotics in Relieving Allergy

*Allergy* is defined as an abnormal response of the immune system to foreign substances called allergens and usually covers food, pollen, fur, wool, drugs, chemicals, animals, dust mites, so forth. Probiotics (Table 2) have been successfully implicated in the prevention of some allergies, including the prevention of atopic dermatitis [38,39,40,41]. Probiotic therapy has also shown positive dynamics in treating rhinitis and allergic eczema by reducing the severity of symptoms. Regarding the treatment of food-related allergies, the results are contradictory and dependent on the specific type of probiotic strain and the duration of treatment [42].

### 3.1. Preclinical Effect

Preclinical treatment of allergies in children is of major importance as it will prevent and decrease the necessity of treatment in later life. Unsuitable access to drugs might also pose an allergic reaction. In this instance, probiotics were proven to be efficient in preventing allergic reactions in early life [45,46,47].

Different *Lactobacillus* spp. strains are known to play a keen role in mediating the immune response due to the appearance of specific suppressive DNA motifs involved in immunity stimulation [48]. During allergic reactions, the stimulation of TLR-9 receptors can interfere with the immune response by hampering Th1 cells; thus, it can overcome the allergy-associated Th2-type immune response. The immunosuppressive motifs (ISMs) can inhibit and deactivate dendritic cells (DC) and preserve Tregs conversion, responsible for the main trigger of allergic cascades [48]. The probiotic bio-active compound, D-tryptophan, isolated from *L. rhamnosus* GG and *L. casei* W56, inhibited the development of allergic airway disease through its capacity to inhibit the production of Th2 cells and chemokines and amelioration of the gut microbial abundance [49]. Interestingly, the biological mechanism of tryptophan was also previously described in conjunction with allergy through the implication in the degradation processes of the immune-regulatory enzyme indoleamine 2,3-dioxygenase (IDO-1), which is consequently enabled by IFN-γ [50]. This process is of major importance as the IFN-γ is a powerful mediator of IDO-1, which in turn degrades essential amino acids as a part of the immunoregulatory pathway to circumvent the immune system over-stimulation. An overactivated immune system can lead to the development can play a role in the pathogenesis of neurodegenerative diseases, the remodeling process in asthma, and lung fibrosis [51].

### 3.2. Clinical Effect

One of the major allergy-related issues in children is related to gastrointestinal reactions to various food products, and probiotics should be part of the clinical tools available for prevention and treatment [47]. It has been shown that administration of probiotics, such as *L. rhamnosus* GG and *B. lactis*, 4 weeks before birth and to the newly born will significantly reduce milk-related allergies, especially when cow’s milk is included in the diet. Moreover, the effect of these specific strains was also extended to a significant improvement in the management of atopic eczema in newly born children. Their positive effects in infants relate to their ability to degrade certain milk and polypeptides and amino acids [4]. Oral administration of *Bifidobacteria* and *Lactobacilli* enhanced IgA production in Peyer’s patches. IgA is known as a potent efficient antibody against conceivably destructive antigens. In a similar vein, *Bifidobacteria* (*B. longum* BB536, *B. infantis* M-63 and *B. breve* M-16 V, and others) spp. have been proven to express similar patterns in decreasing allergic inflammation [52].

One recent human study concluded that a mixture of *Bifidobacteria* was efficient in improving the QOL and alleviating nasal symptoms of children with pollen-caused allergic rhinitis and intermittent asthma [44]. Suggestively such sources of D-type (or bacterial) amino acids isolated from commensal bacteria can provide hints to regulate different allergic reactions within the host [53]. These initial results led to further clinical studies, which showed that strains such as *B. bifidum* BGM4, *B. lactis* AD011, and *L. acidophilus* AD031 can also protect against food-related allergies in children [54,55,56,57,58]. The information presented above clearly shows a connection between the probiotic gastrointestinal mechanisms through the production of SCFA (short-chain fatty acids), which are known for their beneficial effects on the immune system, but, most importantly, for their anti-allergic capacity [4,53]. SCFA can behave as histone deacetylase HDAC inhibitors, up-regulate the expression of the scurfin (FOXp-3) protein acetylation, improve the modulation of innate lymphoid cells via TLR receptors and boost the secretion of IFN-γ and IL-4 [59].

A third clinical effect refers to the effect of probiotics in preventing allergic reactions caused by pollen. It has been shown that *L. acidophilus* NCFM (ATCC 7000396) and *B. lactis* BI-04 (ATCC SD5219) are able to prevent pollen-related eosinophils infiltrations in the nasal mucosa leading to a major reduction in the severity of the allergic reactions [60]. This study indicated that reduction in pollen-related allergies are associated with probiotic-induced changes in fecal microbiota and indicated a trend for reduced nasal symptoms.

## 4. The Possible Role of Probiotics in Mitigation and Regulation of Cancer

Probiotics (Table 3) can inhibit the proliferation of cancer cells, delaying the generation of tumors and preventing deleterious adverse effects linked with cancer treatment [61]. The anticancer activity of probiotics involves various mechanisms, including modulation of the immune system, apoptosis, antioxidant activity, epigenetic mechanism, and DNA damage prevention [62]. Probiotics (e.g., *Bifidobacterium* and *L. acidophilus*) have the ability to decrease the formation of β-glucuronidase and nitroreductase, providing a very efficient mechanism aimed at preventing conversion of procarcinogens into carcinogens.

### 4.1. Preclinical Effect

Currently, a large number of probiotic products are under the preclinical stage to investigate their role in alleviating and treating cancer symptoms [61]. The ultimate goal is the identification of particular probiotic stains or their derived molecules or blends and to develop targeted interventions on the microbiome to assist and improve anticancer treatment and support in alleviating the side effects of existing anticancer therapies (chemotherapy, radiotherapy, and immunotherapy). For instance, a recently pyrosequencing study showed that probiotics decreased the *Fusibacter* genus, which was earlier associated with a contributory determinant of tumorigenesis [68]. Considering this, a piece of new evidence presented probiotic-derived P8 protein that was suggested as a promising candidate for biopharmaceutical interest, which resulted in exhibiting high cytotoxicity in cancer cells via the inhibition of the p53-p21-Cyclin B1/Cdk1 pathway related to hamper the development of the cellular G2 phasic cycle [69]. In addition, a probiotic product resulted in suppressed inflammation and prevention of colon cancer and reduced the expression of COX-2, an enzyme responsible for the catalysis of prostaglandins from arachidonic acid, a marker correlated with increased risk of developing colorectal cancer (CRC) [70]. *Lactobacillus* revealed its anticancer efficiency by releasing nitric oxide responsible for suppressing metabolic pathways in cancer cells and previously has shown a vital role in cervical cancers and the ability to induce an auto-aggregation mechanism that impedes the adhesion of pathogens to vaginal epithelial cells [71]. One representative (*L. casei*) showed to trigger apoptosis and inhibit the tumoral growth via the secretion of a tumor-suppressive cyclic hexapeptide ferrichrome [5]. Literature indicates that the main probiotics that might be involved in the prevention of colon cancer are *L*. *rhamnosus*, *L. kefiri*, *L. salivarius*, *L. acidophilus*, *L. plantarum*, *L. delbrueckii*, *L. casei*, *B. longum*, *B. infantis*, *B. bifidum*, *B. brevis*, *E. coli Nissle* 1917, and *S. thermophilus* [5]. Additionally, the probiotic supplementation with *Clostridium butyricum* reduced the systemic inflammatory responses, alleviated the chemotherapy-induced diarrheal episodes, and sustained the intestinal microbiota in lung cancer patients [72].

The general anticarcinogenic action of probiotics are assigned to the mechanistic insights: the potential to modify gut microbiota composition and the metabolic activity within; the production of specific compounds with anticarcinogenic potential (SCFA and CLA), including their activity to lower the gut pH; the production of antioxidants (SOD, CAT, and GSH); the decrease in biofilm formation via TLR; the ability to inhibit cellular proliferation and induce an apoptotic response in cancer cells; the ability to influence specific carcinogenic and mutagenic factors; the capacity of binding and degradation of carcinogenic molecules from the lumen; the immunomodulatory aspect responsible for the enhancement of the intestinal barrier; and their capacity to promote the production of cytokines, mucin, defensins, and immunoglobulins that confers tissue repairment [64,71,73]. The intestinal microbiota is connected to the development of gastrointestinal cancers due to the generation of genotoxic and toxic microbial metabolic compounds that lead to the formation of mutations through the attachment of specific cell-surface receptors, thereby altering intracellular signal transduction [70]. Specifically, other interesting patterns produced by probiotics are the neutralization and reduction in the absorption of toxic mutagens that can contribute to colon cancer; or the probiotic can lower the expression of bacterial noxious enzymes (e.g., β-glucuronidase, β-glucosidase, nitroreductase, azoreductase, etc.) thereby preventing their conversion from pre-carcinogens into active carcinogenic compounds [71]. Other recent studies had indicated that the human use of probiotic lactic acid bacteria could lower chemotherapy or radiotherapy-induced gastrointestinal toxicity and mucositis without causing any negative effects [4,59,73].

The common anti-inflammatory effects of probiotics arise from the regulation of inflammatory mediators (interferons, interleukins, and cytokines) by controlling the inflammation and carcinogenesis processes [71]. Earlier findings have reported that several probiotics of *Bifidobacterium*, *Lactobacillus*, *Propionibacterium freudenreichii,* and *S. salivarius* were able to synthesize CLA in the terminal ileal segment that could be absorbed or co-interact with colonic cells and induce beneficial outcomes in combating cancer [68]. Additionally, probiotics can release particular chemicals, such as bacteriocins, able to cause leakage of K + ions, amino acids, and ATP from the cytoplasm of invaded cells [74]. Subsequently, bacteriocins induce cellular lysis and prohibit or inhibit the processes of proteins, DNA, and RNA synthesis. For example, probiotic bacteria can secrete metabolites such as lipopolysaccharides (LPS) that activate TLR-4 and trigger immune T-cell-mediated response versus tumoral cells [61]. Extracted exopolysaccharide (EPS) from *L. acidophilus* 20,079 was previously demonstrated to have direct cytotoxicity against tumoral cells through mechanistic traits such as immune response stimulation, apoptosis, and inactivation of NF-κB inflammatory signaling pathway [61]. Several clinical oncology studies have indicated that the mitigation of human colon cancer occurs due to the possibility of probiotics to deactivate and detoxify mutagenic compounds and the potential to provide antimutagenic substances that will reduce the assimilation of mutagens and proliferation of carcinogenic bacteria by subsequently improving immunological functions [4,61]. The general consumption of traditional fermented foods with sufficient concentrations of bacterial or yeast probiotics may decline the risk of CRC development, including other chronic illnesses [70].

### 4.2. Clinical Effect

In a recent randomized, double-blind placebo study, it was concluded that the probiotic oral therapy by six viable strains (*Lactobacillus* and *Bifidobacteria*) for 6 months was demonstrated to significantly affect the expression of pro-inflammatory markers (IL-6, IL-10, IL-12, IL-17A, IL-17C, IL-22, and TNF-α) in CRC subjects, also within the study the treatment did not aggravate diarrheal frequency and canceled the necessity of antibiotic therapy [63]. Similarly, the administration of a probiotic product combined with omega-3 fatty acid showed to notably enhance the QOL index, decrease the levels of IL-6, and weakened the negative effects of chemotherapy in CRC patients in comparison to the control placebo group in which a significant increase in TNF-𝛼 and IL-6 was observed [64]. Earlier randomized, double-blind, placebo human studies confirmed that pre-surgical dietary supplementation with probiotics promotes the restitution of normal gut function associated with a faster recovery in patients subjected to CRC surgery [65]. Previously, in gastric cancer patients who followed the surgery practice with gastrectomy, flowed by oral probiotic blend (*B. infantis*, *L*. *acidophilus*, *E. faecalis*, and *B. cereus*) supplementation remarkably boosted immune response (lymphocytes) and decreased severity of inflammation (leukocytes) via modification of GIT microbiota composition [66].

Recent clinical data suggest that probiotics can also play a significant role during the recovery stage following surgery for gastric cancer. It has been shown, in clinical studies, that probiotic strains such as *L. plantarum* MH-301 (CGMCC NO. 18618), *L. rhamnosus* LGG-18 (CGMCC NO. 14007), *L. acidophilus*, and *B. animalis* subsp. lactis LPL-RH (CGMCC NO. 4599 could significantly lower postoperative inflammation, enhance immunity, resume gut microbiota composition and promote postoperative recovery. All these effects lead to the conclusion that probiotic compounds can restore gut microbiota homeostasis, reduce inflammation, maintain intestinal mucosal barrier and immunity, finally promote recovery after gastrectomy, and are anticipated to improve the prognosis of patients [75].

The hypothesis that specific probiotic strains may manipulate cancer-associated microbiota and nourish its ecology with beneficial bacteria was also supported by other researchers that observed significant induced differences in tumoral microbiota patterns in CRC patients that received probiotics in comparison with control microbiota [67]. It was shown how many beneficent effects probiotics can harbor that could further act as cancer alleviators, protectors, and regulators. Based on the results gathered from in vitro, in vivo, and clinical trials, the effects are promising; however, there are still many limitations, especially in the human trial quarter [76].

## 5. The Pre and Clinical Neurological Effect of Probiotics

Probiotics bearing the potential to modulate and impact the central nervous system (Table 4) and consequently influence mood, anxiety, and cognitive functions via bidirectional neuronal signaling pathways and are denoted as “psychobiotics” [77]. However, nowadays, the denomination of psychobiotics encompasses all microbiota-assisted implications, including probiotics and prebiotics that could potentially interfere with the bacteria-brain relationship and provide mental health benefits to the host [10]. Different bacteria-derived compounds (e.g., low molecular compounds, proteins, peptides, cell wall components, etc.) with neuroactive potential can behave as potential mediators between bacteria and host [77]. Probiotics can synthesize strain-specific neuro-metabolites such as dopamine, gamma-aminobutyric acid (GABA), SCFA, serotonin, biogenic amines, amino acids, phenolic compounds, organic acids, which are known to provide adjuvant ameliorative effects for the management of several neurological/psychical disorders (e.g., depression, anxiety, stress) through the gut-brain axis pathway [78]. Several disorders emerging from brain inflammatory processes are interlinked with the gut microbiota composition. The exact possible biological mechanisms by which probiotics communicate with the microbiota-gut-brain axis are still not well understood; however, significant progress has shown that the main mechanistic insights involve endocrine, neural, and immune signaling pathways [10]. Moreover, the modulation of the gut-brain axis through probiotic application opens a novel frontier window inclined toward mental disorders management and prevention, expanding probiotic efficiency in chronic diseases aside from their established key roles within the gut disorders.

### 5.1. Preclinical Effect

The involvement of probiotics in alleviating neurological disorders is gaining more and more interest, especially due to the recent data originating from preclinical studies. Attempts have been made in preclinical to study the human inflammatory demyelinating disease, also known as multiple sclerosis (MS), using the experimental autoimmune encephalomyelitis (EAE) mouse model [85]. Combinations of *L. paracasei* DSM 13434, *L. plantarum* DSM 15,312, and *L. plantarum* DSM 15,313 were identified capable of suppressing the progression of EAE and reverse the disease by down-regulating MOG-reactive T-cells [86]. In addition, in mice, similar combinations of *S. thermophilus*, *L. reuteri*, *B. bifidum*, *L. acidophilus,* and *L. casei* (1 × 10^8^ CFU for each strain) were able to significantly decrease the occurrence of EAE, clinical scores, and inflammation of the spinal cord [87]. In a more recent preclinical study, it was determined that colonization with the probiotic bacterium *E. coli* strain Nissle 1917 (EcN) was an efficient prophylactic strategy aimed to prevent EAE. The authors have described those two doses of EcN delayed disease occurrence and its severity. Moreover, they have shown that the EcN-treated mice had decreased amounts of perivascular cuffing, CD4+ T-cell infiltration, and significantly decreased Th1 cells levels coupled with reduced activation of microglia [88].

The autism spectrum disorder (ASD) has been described as the inability to properly contextualize information and is recognized as a critical dimension of social cognition [89]. Oral administration of *B. fragilis* (1 × 10^9^ CFU) in the maternal immune-activation model of ASD in mouse offspring resulted in improved anxiety-like, stereotyped, sensorimotor, and communicative behaviors indicating the microbial supplement a possible treatment in ASD [90]. Combinations of *B. longum*, *B. breve*, *B. infantis*, *L. helveticus*, and *L. rhamnosus* had a positive effect in alleviating ASD symptoms in studies performed either in animals or humans [91].

Inflammation of the peripheral and central nervous system leads to the classic symptoms of Alzheimer’s disease (AD) [92]. A recent study identified that the gut microbiome has a significant impact on the disease pathology in an AD mouse model (5xFAD). Inclusion of *L. acidophilus* and *L. rhamnosus* in the drinking water of the 5xFAD mice transiently increased *Lactobacillaceae* species without improving the nesting score but displayed reduced plaque load in the hippocampus [93]. In a similar mouse model, *L. delbrueckii* was described as able to improve depression-like behavior through inhibiting toll-like receptor 4 (TLR4) [94]

### 5.2. Clinical Effect

Probiotics can regulate the neurotransmitters and proteins, including gamma-aminobutyric acid (GABA), serotonin, glutamate, and brain-derived neurotrophic factor (BDNF), involved in mediating the neural excitatory-inhibitory balance, mood, cognitive functions, learning, and memory processes [95,96,97]. Several reports revealed that *Lactobacillus* and *Bifidobacterium* species (*L. plantarum* 90sk, *L. brevis* 15f, *B. adolescentis* 150, and *B. angulatum* GT102) produce Gamma-aminobutyric acid (GABA) [62]. When GABA is connected to the specific proteins (GABA_A_ and GABA_B_) within the brain, it creates an effect that can assist in regulating the disorders associated with anxiety, fear, and depression. In addition, *Bifidobacterium*, *Lactobacillus*, and *Faecalibacterium* previously showed a positive correlation with a healthy brain status by conferring resilient effects against neurological disorders [98]. Specifically, *B. adolescentis* can exhibit anti-depressive and anxiolytic behavior by hampering inflammatory cytokine release and the potential of reshaping the gut microbiota [99], and collectively such probiotic metabolites reflect a neuroactive capacity of gut microbiota involved in sustaining the normal functionality of the nervous system [77]. A recent human randomized, pilot 90-day trial aimed to evaluate the safety and efficiency of probiotic strain *B. coagulans* MTCC 5856 on the amelioration of major depressive disorder symptoms in patients suffering from irritable bowel syndrome (IBS) detected significant observations [79]. A dosage of 2 × 10^9^ CFU spores/day resulted in improving depression clinical scoring parameters, notably at the same time reducing the symptoms associated with irritable bowel syndrome in comparison to the placebo group. Moreover, the inflammatory biomarker, serum myeloperoxide, was also significantly decreased in patients receiving probiotic treatment. However, due to the co-diagnosis of the patients with IBS, it is hard to draw a definite conclusive statement. Likewise, an 8-week double-blind clinical investigation demonstrated that oral supplementation with the probiotic product (*L. helveticus* R0052 and *B. longum* R0175) significantly decreased depression inventory score, including kynurenine/tryptophan ratio and elevated tryptophan/isoleucine ratio in probiotic-assisted patients compared to placebo treatment [80]. It was also suggested that reduction in the kynurenine/tryptophan ratio might indicate one of the possible probiotic-mediated mechanisms for depression alleviation. Likewise, in the most recent 8-week open-label pilot study, the author concluded that dietary addition of *L. helveticus* R0052 and *B. longum* R0175 at 3 × 10^9^ CFU/day mitigated the symptoms of depression in treatment of moderately depressed subjects [81]. However, due to the study’s limitations, such as the short sample size, it is irrelevant to express robust conclusions.

Another recent randomized, triple-blind, placebo-controlled trial reported the remarkable enhancement in cognitive reactivity index status (LEIDS-R) of depressed patients after receiving the commercial probiotic product (*B. bifidum* W23, *B. lactis* W51, *B. lactis* W52, *L. brevis* W63, *L. casei* W56, *L. lactis* W58, *L. salivarius* W24, *L. acidophilus* W37, and *L. lactis* W19) prescribed at 1 × 10^10^ CFU/day, while no significant findings where seen in depression, stress and anxiety parameters [82]. LEIDS-R is associated with a bad mood and is the main predisposal factor to depression. Moreover, in previous clinical trials, dietary inclusion by the same product was concluded to help patients by relieving migraine occasions [83]. Additionally, probiotic consumption (*L. plantarum* P-8) was recently postulated to improve anxiety and stress symptoms in stressed individuals via the modulation of gut microbiota interlinked with neuroactive potential [84]. A twelve-week supplementation enriched the diversity of neuro-transmitting synthesizing/consuming species-level genome bins and expanded the levels of microbial metabolites with neuro-active capacity (such as SCFA, GABA, sphingomyelin, and arachidonic acid), respectively. These findings highlight a possible connection between stress, anxiety, and probiotic provoked gut microbiota modulation (gut-brain axis) that might involve coping with anxiety, stress, and depression disorders.

Although the abundant evidence involving animal models describes the anti-depressive, antioxidant, and neuro stimulating efficacy of probiotics/psychobiotics and their metabolites activity on mental health via behavioral tests, the vast majority of those results are not reproducible in humans [77]. Moreover, the anti-depressive efficiency of probiotics has not been validated for human clinical trials since there are only a bunch of studies available [4]. Furthermore, there is a crucial requirement for a conclusive confirmation to evaluate probiotic action on the gut-brain axis involving clinical trials on humans. The therapy using psychobiotics to combat different neurological disorders or prevent them by probiotic intervention could be a sustainable topic in the future.

## 6. Probiotics Involved in Obviation of Alzheimer’s Disease Progression and Amelioration of Cognitive Conditions

Aging constrains longevity and leads to the establishment of neurodegenerative disorders. The perturbation of the gut microbiota is established in different neuropsychiatric disorders and is commonly associated with dementia diseases such as Parkinson’s, Alzheimer’s, and others [100,101]. With the aging process, various bacterial species (e.g., *E. coli*, *S. aureus*, Salmonella, *K. pneumonia*, *Mycobacterium*, and *Streptococcus*) within the gut can generate amyloid proteins that could multiply the production and accumulation of amyloid-beta (A-β) plaques that result in the aggravation of Alzheimer’s disease (AD) [102,103]. Today there is no definitive treatment against this type of incurable dementia, and the authentication of novel alternative therapeutic agents is of great interest to tackle this problem [101].

During the pathogenesis of AD, there are numerous involved factors in the disease progression, and the principal hallmarks are commonly linked with the overproduction of ROS that induces neuroinflammation and consequently cellular death, the progression of A-β plaques, peptides and oligomers, microglial activation, neurofibrillary tangles, mitochondrial damage and vascular abnormalities [100,104]. Afflicted lipid metabolism could trigger neurodegenerative processes, and reversely Labarre et al. have shown that dietary treatment with *L. rhamnosus* HA-114 restored lipid homeostasis, including energy balance via mitochondrial b-oxidation [105]. According to previously documented studies, probiotics have shown impressive ability to decelerate AD advancement due to clinically proved anti-inflammatory and antioxidant effects, including their ability to normalize cognitive deterioration that appreciably highlights their therapeutic applicability and effectiveness [106,107,108,109].

Of late, studies have suggested that probiotics could shape and induce balance in the microbiome and ameliorate AD symptoms via miscellaneous mechanisms [106]. Most of these mechanisms are generally assigned due to the production of antioxidants and their potential to neutralize ROS and inhibit the cytokine production of pro-inflammatory cascades [106]. Another mechanism of interest is ascribed to the potential of strengthening the epithelial barrier in order to defend the enteric nervous system (ENS) against possibly toxic compounds. Other possible mechanisms of how probiotics can protect the brain against neurological disorders (AD) rely on the bilateral/bidirectional communicative potential of how the microbiome interacts with CNS and ENS mediated through the microbiota-gut-brain axis [110]. For example, dysbiosis in elderly persons could cause a leaky gut syndrome that can trigger a silent cascade of neuroinflammatory pathways, and the key pathomechanism occurs via bottom-up signaling and is considered a major risk factor for AD development [111,112]. The bottom-up signaling pathway implicates the relocation of pro-inflammatory mediators from GIT straight to the brain through different migration routes such as blood circulation, microbial metabolites, vagus nerve, immunity, and ENS [113]. In fact, the second-largest proportion of the neuronal cells within our body is localized in ENS. It is accountable for essential functions (e.g., mucus secretion, angiogenesis, stability of the blood flow homeostasis and motility) of GIT. The vagus nerve moderates and synchronizes information exchange amid the brain and the GIT via dopamine, serotonin, glutamate and GABA neurotransmitters.

The probiotic mechanism of immunomodulation for ameliorating AD symptomatology is speculated to be exerted by regulating inflammatory processes evoked by A-β deposition and other factors such as obesity, brain injury, and inflammaging [109]. Meanwhile, SCFA produced by probiotics such as butyrate and propionate can also improve AD through a mechanism that modulates the synthesis of neurotransmitters able to normalize neurotrophic genes viz nerve growth factor (NGF) and brain-derived neurotrophic factor (BDNF), respectively [109]. The positive impact of butyrate was previously associated with anti-inflammatory and immuno-modulating properties. Both are relevantly important effects for elderly subjects prone to undergo immune-senescence and develop different physiological disorders, including infections [114]. In the pathogenesis of AD, the kynurenine metabolic pathway (KP) is involved in the tryptophan catabolic processes that play a major role in regulating serotonin bioavailability [102]. The metabolic products from tryptophan metabolism could contribute to oxidative stress in neurons, cellular death and promote tau phosphorylation and neurofibrillary tangle formation [103]. Abnormalities of the regulation of kynurenine and serotonergic pathways in tryptophan metabolism affect the pathological state of CNS, such as dementia, AD, and Huntington’s disease [102].

Interestingly probiotics can change and normalize tryptophan and *kynurenine* levels favorably for the host by enhancing cognitive functions [115,116,117]. Impairment in the serum and brain expression of the blood BDNF signaling may play an important role as a viable indicator or a confounding factor in neurodegenerative processes, including AD pathogenesis [107,118]. In contrast, probiotic supplementation with probiotics such as *B. bifidum* BGN4 and *B. longum* BORI significantly raised serum BDNF levels, meanwhile displaying a negative correlation with gut bacteria (*Eubacterium* and *Clostridiales*) in elderly patients [107]. The increased expression in BDNF was suggested to augment brain functionality. In addition, compared with healthy subjects, patients suffering from AD contains a 3-fold elevation of the serum LPS concentrations that could cross the blood-brain barrier through the interaction with specific CNS receptors (CD14) and TLR4 facilitating the progression of innate immune response accompanied by neuroinflammation and cellular death [106,119]. Although the use of probiotics to prevent different types of dementia has numerous limitations, mounting evidence revealed that manipulations of the gut microbiota via probiotic strains could be a new alternative option to stop, hamper and even prevent AD in prodromal instances [100,106].

### 6.1. Preclinical Effect

Several authors have reported the anti-Alzheimer activity of probiotics in several in vivo murine models [110,120,121,122,123,124]. These findings were echoed in a previous study where the authors showed that *L. plantarum* MTCC 1325 might induce ameliorative outcomes in D-Galactose-induced AD in rats [120]. Histopathological brain examination revealed that rats supplemented by probiotics had healthier neuronal conditions, presenting small degenerative partial processes reaching 30 days of treatment; nevertheless, after 60 days, a visible recovery was observed. Interestingly, the AD group that has not received probiotics displayed beta-amyloid (β-amyloid) plaques accompanied by neurofibrillary tangles in the hippocampus and cerebral cortex. In addition, the *L. plantarum* notably raised the levels of acetylcholine (ACh) concentration in the hippocampus and cerebral cortex, a characteristic suggested in the overlapping of bidirectional communication and antioxidant capacity of *L. plantarum* MTCC 1325. Taken together, the authors have concluded that *L. plantarum* MTCC 1325 exerts anti-Alzheimer activity by enhancing behavioral activity, learning skills via upregulation of cholinergic neurotransmitters of the brain, increasing the body weight, and reversing histopathological injuries similar compared to the control group [120].

In another study, mice that received intracerebroventricularly Aβ25-35 protein for AD activation and then treated with *B. breve* A1 were suggested to obviate cognitive-induced deterioration [122]. The authors specified that daily supplementation by *B. breve* A1 showed to diminish alternation behavior impairment on a par with cholinesterase inhibitor donepezil, regulating to the similar values detected in the control animals. Solely, the injection with Aβ25-35 depicted a reduction in alternation behavior and was linked with abated working memory. Other observations indicated that probiotic administration could modulate the immune response by mitigating the toxicity of A-β as well boosted the concentration of plasma SCFA in acetate after following Aβ25-35 injection [122]. The authors stated that *B. breve* A1 exerted the protective effects probably via increased production of acetate.

Lately, *Bonfili* and co-workers have investigated the live probiotic formulation SLAB51 (*S. thermophilus*, *B. longum*, *B. breve*, *B. infantis*, *L. acidophilus*, *L. plantarum*, *L. paracasei*, *L. delbrueckii* subsp. *bulgaricus*, and *L. brevis*) against brain-type oxidation related to AD [110]. The authors used suitable transgenic 3xTg-AD mice possessing A-β intracellular immunoreactivity by reflecting the model associated with AD patients. First of all, the treatment with probiotic mixture induced remarkable increases in the expression and activity of mice brain Sirtuin-1 (SIRT-1), an enzyme correlated with robust antioxidant and neuroprotective effects that can regulate the expression of antioxidant biomarkers such as CAT, SOD, PRDX-3, and PRDX-5 [110]. Secondly, the treatment greatly alleviated the drop in SIRT-1 levels and was inversely correlated with p53 acetylation, indicating the mitigative properties of SLAB51 toward p53-mediated apoptotic events. Impaired expression of SIRT-1, plus the agglomeration of tau and A-β in the cerebral mantle, strikingly impacts the health conditions in subjects suffering from AD. Relevant reduction in pro-inflammatory cytokines, including biochemical parameters such as GPx, was counteracted by the probiotic treatment as well as lipid oxidation parameters 4-hydroxy-2-nonenal (4-HNE), nucleic acid oxidation indices such as 8-oxoguanine DNA glycosylase-1 (OGG1) and 8-hydroxy-2′-deoxyguanosine (8-oxodg) levels were partially and remarkably restored in AD animals subjected to probiotic mixture compared to the control group. In conclusion, their study demonstrated the positive impact of SLAB51 on the SIRT-1 reactivation that prevents the animal brain from oxidation and maintains redox homeostasis while improving AD symptoms. Of note, SLAB51 also showed the ability to delay Parkinson’s disease progression in the mice model by defending dopaminergic neurons, regress neuroinflammation and oxidative stress [125]. Similarly, an earlier exclusive report showed that SLAB51 formulation restores damaged neural pathways such as ubiquitin-proteasome system and autophagy in 3xTg-AD mice [124]. Furthermore, the probiotic elevated the abundance of *Bifidobacterium* spp., meanwhile presented to decrease the abundance of *Campylobacterales*. The treatment of AD mice with SLAB5 fortified the SCFA profile by greatly increasing the levels of propionic, acetic, and butyric acids, alongside provided remarkably decrease Aβ1-42 load and resulted in attenuating of the expression of pro-inflammatory cytokines such as IL1-α, IL1-β, IL-2, IL-12, IFN-ɣ, and TNF-α, respectively [124]. Another team of researchers recently reported the affirmative effect of probiotics on synaptic plasticity in β-amyloid infected Wistar rats. In addition, the probiotic (*L. acidophilus*, *B. bifidum,* and *B. longum*) supplementation significantly reduced malondialdehyde (MDA) levels, normalized the antioxidant capacity (TAC), and efficiently restored long-term potentiation (LTP) and field excitatory postsynaptic potentials (fEPSPs) in the hippocampus compared to other groups [123]. Furthermore, in a recent work, it was demonstrated that probiotics (*L. acidophilus*, *L. fermentum*, *B. lactis*, and *B. longum*) modulated microbiota by enhancing memory deficit and suppressing the pathomechanisms induced by Aβ1-42 AD rats [126]. The injection with a dose of Aβ1-42 triggers the plaque generation, shape abnormalities of cells and memory and learning impairment. Overall, the results of such probiotic treatment ameliorated oxidative stress parameters, causing the reductions of MDA from hippocampal tissue sight and aligned the antioxidant profile compared with the control group. Finally, the authors suggested that such treatment results and observations could potentially lead to the clearance of plaques [126]. Overall, these findings described mechanistic insights highlighting the efficacy of probiotics in the role of modulators of inflammatory processes, which led to the amelioration of AD pathology and symptomatology in animal models.

### 6.2. Clinical Effect

So far, clinical studies involving probiotics and their declining effects on human AD progression are scarce and limited. However, we will describe in this sub-chapter the recent and existing therapeutic interventions by probiotic supplementation targeted to counteract AG progression in elderly patients (Table 5).

The results of the recent meta-analysis supplied confirmation that sufficient probiotic supplementation over a longer period of time might enhance cognitive function [132]. Within this context, a prolonged 12-week administration of 200 mL/day of milk infused with probiotics (*L. acidophilus*, *L. casei*, *B. bifidum*, and *L. fermentum*) in a concentration of 2 × 10^9^ CFU to the patients with AD, significantly (*p* < 0.001) improved mini-mental state examination (MMSE) score, a test for measuring cognitive criteria in human subjects [128]. Additionally, the plasma MDA and hs-CRP levels also showed significant decreases (*p* < 0.001) along with the remarkable reductions in homeostatic model assessment for B-cell function (HOMA-B) index compared with control AD subjects without probiotic supplementation. Similarly, in the open-label, single-arm study, *B. breve* capsule supplementation at the concentration of 2.0 × 10^10^ CFU significantly elicited the MMSE score in AD patients following 24 weeks of treatment [127].

Lately, a probiotic aqueous formulation containing *L. casei* W56, *L. lactis* W19, *L. acidophilus*, W22, *B. lactis* W52, *L. paracasei* W20, *L. plantarum* W62, *B. lactis* W51, *B. bifidum* W23, and *L. salivarius* W24 (Omnibiotic Stress Repair), was proclaimed to greatly lift serum kynurenine concentrations, influence serum tryptophan metabolism and immune homeostasis in AD patients [112]. Meanwhile, Leblhuber et al. specified that significant increases in serum kynurenine levels are possibly triggered by macrophage activation identified through the correlation between Δ-Kyn/Trp and Δ-neopterin concentrations [112]. Their results showed that probiotic mixture positively affected gut microbiota composition that caused a reduction in fecal zonulin expression and raised the abundance of *Faecalibacterium prausnitzii* in AD patients in contrast to baseline. The increased zonulin expression acts as an inflammatory biomarker that indicates the impairment of gut barrier functions (leaky gut) and tends to increase rapidly in elderly patients. In contrast, Rudzki et al. observed that for elder patients suffering from major depressive disorders, which received *L. Plantarum* 299v capsules led to a reduction in kynurenine concentrations [117]. Their treatment also raised the 3-hydroxykynurenine: kynurenine ratio, which was deduced to augment their cognitive functions compared to the placebo control group.

A recent randomized, double-blind, controlled trial probiotic (*L. acidophilus*, *B. bifidum*, and *B. longum*) at the concentration of 2 × 10^9^ CFU/day co-supplemented with selenium 200 mg/day showed to improve the features of metabolic profile and refine cognitive functions [129]. Their results displayed that combinatorial 12 weeks treatment appreciably enhanced MMSE, significantly reduce insulin concentrations and slightly increase quantitative insulin sensitivity check index (QUICKI) compared with placebo and solely selenium group. Furthermore, the authors noted remarkable alleviations in serum hs-CRP, LDL, including the total/HDL cholesterol and triglyceride levels. Meanwhile, the concentrations of TAC and GSH presented significantly increases. Finally, probiotic and selenium administration appeared to regulate and efficiently improve the gene expression of TNF-α, PPAR-γ, and low-density lipoprotein receptor (LDLR) [129].

According to the recent findings of *Ton* and collaborators, a formula with fermented milk with kefir grains containing probiotics *Acetobacter aceti*, *Acetobacter* spp., *L. delbrueckii*, *L. fermentum*, *L. fructivorans*, *E. faecium*, *Leuconostoc* spp., *L. kefiranofaciens*, as well as yeast cultures of *Candida famata*, and *Candida krusei comprising the total* CFU of 7.5 × 10^7^ and supplemented at the concentration of 2 mL/kg/daily demonstrated potentiated effects in elderly patients with AD by inducing repairing outcomes on cognitive dysfunction, global cognitive status, showed to improve the memory, visual-spatial and abstraction capacities, executive language and constructive abilities [130]. In addition, systemic inflammation and oxidative stress, including blood cell damage, were comparatively abolished. The overall probiotic 90 days treatment, presented to reduce the pro-inflammatory cytokines associated with the pathogenesis of neurodegenerative disorders (TNF-α, IL-8, and IL12-p70), including (IL-8/IL-10 and IL-12/IL-10) ratio. Likewise, the expression of advanced oxidation protein products (AOPP), an oxidative stress biomarker alongside serum concentrations of O^2−^, H_2_O_2_, and ONOO^−^/OH^−^ substantially decreased. At the same time, the levels of NO significantly increased at the end of the treatment. The sophisticated parameters reflecting mitochondrial dysfunction notably decreased, while mitochondrial integrity and the indices for preserving mitochondrial function increased following kefir treatment. In a novel way, the blood expression of apoptotic and cell cycle arrest regulator p53 was elevated, DNA fragmentation and hallmark of apoptosis poly (ADP-ribose) polymerase 1 (PARP-1) declined in a statistically significant manner. Their study has concluded that such adjuvant therapy could mitigate systemic inflammation, blood cell damage, oxidative stress, and adjust cognitive deficits against AD progression [130]. Finally, the perioperative oral administration of probiotic (*B. longum*, *L. acidophilus,* and *E. faecalis*) capsules at the dosage of <1.0 × 10^7^ CFU in elderly subjects was reported to prevent postoperatively cognitive deterioration, probably through the interfering potential to impede stress response, including peripheral inflammatory processes [131].

## 7. The Impact of Probiotics on Longevity, the New Concept of “Gerobiotics”

Alongside the discussed possible effects of probiotics against the mitigation of respiratory infections, cancers, potential to relieve allergies, neurological disorders, including Alzheimer the ongoing scientific progress is focused on the novel properties of probiotics to expand the lifespan. Thence, this section aims to briefly describe probiotics’ role and their mechanistic features in influencing fundamental aging processes associated with longevity.

Nowadays, the changes of *Homo sapiens* microbiota are expected to exert an ultimate impact on aging advancement [133]. It is speculated that probiotics can blunt the progression of aging processes through the possible factors that govern age-related changes of the gut microbiome, age-related hallmarks (e.g., mitochondria dysfunction, telomere attrition), impact the severity and progression of cellular senescence, oxidative stress, epigenetic alteration, alteration of intracellular and intercellular communication, deprivation of proteostasis and including others [133,134,135]. For example, *Kumar* and co-workers recently reported the anti-senescence potential of secretory metabolites of *L. fermentum* that mitigated the formation and severity of stress-induced senescence by a mechanism that relied on the downregulated phosphorylation of the PI3K–Akt–mTOR pathway and modulated the senescence-associated biomarkers such as p21WAF1, p38MAPK, p53, SA-β-gal, iNOS, COX-2, NF-κB and DNA damage response in 3T3-L1 preadipocytes [136]. However, these remarks were claimed and observed only in several in vitro and in vivo studies, excluding human subjects [137,138]. At the moment, testing probiotic bacteria for longevity advancement in humans is not reliable due to comprehensive and complex study design, which would require decades of years for investigations, data analysis, and reproducibility to wrap up at least a definitive small-scale conclusion. Recently, a group of researchers defined probiotics that can prolong the health span of the host as “gerobiotics” [133]. The use of probiotics to attenuate the mechanisms of aging and minimize physiological aging processes is a new concept of major importance but will require decades of years to unveil. Initially, it is essential to unlocking a vast array of possible molecular mechanisms of “gerobiotics”, followed by in vitro, ex vivo in vivo till clinical investigations, which together would require the utmost sources of funding for conceptual realization.

So far, significant progress regarding deciphering the increasing- longevity mechanisms of prebiotics has been made on worm (*Caenorhabditis elegans*) models [138,139,140,141,142,143,144]. The *C. elegans* model is a suitable, fast, and predictive screening tool for identifying novel molecular mechanisms. In a very recent genomic analysis, Yu et al. isolated a novel *L. plantarum* FLPL05 strain from the stool of the elder subject with increased longevity patterns from Gaotian Village of China and demonstrated to possess remarkable antioxidant activity in a murine model; however, longevity and lifespan outcomes were not assessed by the authors [145]. Intriguingly, in a study labored by Zhao et al., the lifespan of worms was enlarged by 28% after feeding with *B. longum* BB68 strain isolated from centenarians living in Bama county of China [138]. The authors showed that the main mechanism responsible for longevity lies in activating the TIR-1–JNK-1–DAF-16 signaling pathway and mediation of cell wall components. The treatment with *B. longum* BB68 presented to greatly increase the expression of SOD-3 and DAF-16 activity that is associated with preserved immune signaling cascade. Similar findings were mirrored in an earlier study when *L. rhamnosus* CNCM I-3690 protected nematodes by lifting their lifespan by 20% and viability by 30% following oxidative stress induction with H_2_O_2_ (3–5 mM) [146]. Moreover, these observations correlated with the DAF-16/insulin-like pathway expression, which is greatly conserved in humans. A recent study concluded that *B. infantis* ATCC 15,697 elicited longevity and improved leaving behavior via TLR/TOL-1 regulation compared with control and mutant worms [144]. Likewise, *E. faecium* L11 isolated from the chicken cecum was recently involved in lifespan extension and inducing protective effects against the damage caused in nematodes after *Salmonella Typhimurium* infection [142]. The results showed that *E. faecium* L11 significantly up-regulated aging and host defense gene expression (e.g., pmk-1, sek-1, dbl-1, and sma-3) compared with the worms fed with *E. coli* OP50 (standard feed for worm). Similar discoveries were recently reported by Wang et al. when *Weissella confusa* CGMCC 19 was administered to *C. elegans* and provided a strong antioxidant potential, prolonged lifespan, and increased their host defense against *salmonella*-induced infection [141]. Kato et al. concluded that *Clostridium butyricum* MIYAIRI 588 (CBM 588), used as an anti-diarrheal agent in Japan, improved nematodes’ lifespan probably via the mechanisms relied on DAF-16/FOXO and the SKN-1/Nrf-2 transcription factors [140]. Afterward, the treatment with CBM 588 resulted in regulating of insulin/IGF-1 signaling (IIS) pathway and the Nrf-2 factors. Previously, *L. gasseri* SBT2055 effected the anti-aging effects in the *C. elegans* model. It was suggested to be executed through a mechanism that upregulates the skn-1 gene expression, including target genes of SKN-1 through the p38 MAPK signaling pathway, which congruently led to an enhanced antioxidant defense and strengthened innate immune response [139]. Another mechanism associated with lifespan extension was recently reported in *Butyricicoccus pullicaecorum* and *Megasphaera elsdenii* that inhabit the human intestinal tract [143]. Their study observed strong interconnection within innate immunity and longevity and suggested that these combinatorial effects occur via activation of a nuclear receptor signaling scaffold as well as the innate immune system in a TGF-β pathway-dependent route but IIS pathway-independent mode, associated with anti-inflammatory processes.

Compared with nematode models, an aberrant progression of life-extending probiotics was mentioned for other in vivo models. A decade ago, an intervention with *B. animalis* subsp. *lactis* LKM512 in an 8-month Crj: CD-1 retired mice model displayed to promote longevity by inhibiting of chronic low-grade inflammation that also showed regulation of intestinal lumen and colon tissular environments compared with the control probiotic untreated animals [147]. The authors speculated that life-extending properties might be attributable to the suppression of the mTOR pathway. Another work executed on D-galactose-induced aging mice showed that dietary administration of *L. paracasei* PS23 precluded muscular dysfunction and age-related muscle loss [137]. The authors specified that attributed ability is exhibited possibly through the mechanistic insight responsible for ghrelin stimulation in the gastric cells. Last but not least, the successful identification of novel probiotic bacteria involves interdisciplinary approaches and technological updates in order to promote them as a new adjuvant therapy. Fundamentally, the application of life science, biotechnology, molecular biology, pharmacology, and genetics in a whole integrated ensemble may play a valuable role as a key tool in developing novel longevity extending probiotic formulations.

## 8. Conclusions

Overall, this review discussed the significant role of probiotics in pre and clinical cases of respiratory infections, cancer, and allergies and also in neurological disorders, highlighting the gaps and the need for future research. Knowledge of microbiome-host interactions paved the way and revealed a new alley of understanding of the immunopathological basis of many disorders. The inoculation of probiotics into foods or supplements can help to maintain a balanced immune system, and it has been shown, in human clinical studies, that probiotics (e.g., *L. Bifidobacterium*) can reduce the incidence of symptomatic illness and the duration of viral infections. These observations were also confirmed in animal models, in which case preclinical observations indicated the ability of probiotics to modulate the immune system and reduce the risk and course of respiratory tract infections [148].

However, further clinical research is necessary to better understand how and why probiotic microorganisms positively affect human health and how their presence within the microbiome can shape its function in improving our understanding of their biological effect.

## Figures and Tables

**Table 1:** Probiotics associated with treatment of viral infections.

Probiotic	Condition	Concentration	Evidence	Ref
*B. subtilis* and *S. faecium * (Medilac-DS)	Pneumonia	1 × 10^9^ CFU	In human trials, improved clinical symptoms and normalized inflammatory biomarker levels (hs-CRP) in patients with pneumonia	[16]
*L. acidophilus* DSM 32241, *L. plantarum* DSM 32244, *L. helveticus* DSM 32242, *L. brevis* DSM 27961, *L. paracasei* DSM 32243, *B. lactis* DSM 32246, *B. lactis* DSM 32,247, and *S. thermophilus* DSM 32345	COVID-19	2.4 × 10^10^ CFU	In human trials, improve clinical symptoms, such as remission of diarrhea, reduces mortality, and decreased the risk of developing respiratory failure	[17]
*L. rhamnosus* HN001, *L. acidophilus* DDS-1 and *B. lactis* Bb-12, and *S. thermophilus*	Upper respiratory tract infection	10^10^, 5 × 10^10^, and 10^7^ CFU	Human trials, indicating a reduction in respiratory tract infections improved quality of life	[18,19]
*L. lactis* JCM 5805	Influenza and common cold	10^11^ CFU	In healthy young adults, decreased the incidence, reduced the number of days of symptoms associated with influenza and common cold through the activation of plasmacytoid dendritic cells and upregulation of IFN-related genes	[20]
*L. delbrueckii* subsp. *bulgaricus* OLL1073R-1	Influenza A virus subtype H3N2	1.8–3.5 × 10^8^ CFU	In human elderly patients improved immunity, prevented infection with influenza A virus subtype H3N2 via the production of influenza A virus subtype of H3N2-bound salivary IgA	[21]
*L. paracasei* N1115	Acute upper respiratory tract infections	3.6 × 10^7^ CFU	In human elderly and middle-aged patients reduced the risk of acute upper tract infections via T-cell natural immune defense stimulation	[22]
*L.rhamnosus* GG, live *B. subtilis*, *E. faecalis*, *B. breve Yakult*, *L. casei Shirota*	Ventilator assisted Pneumonia	2 × 10^9^, 4.5 × 10^9^ and 0.5 × 10^9^, and 1 × 10^8^ CFU	Human trials indicated substantially less incidence of ventilator-associated pneumonia	[15,23,24]

**Table 2:** Probiotics associated with attenuation of allergic reactions.

Probiotic	Condition	Concentration	Evidence	Ref
*L. rhamnosus* *B. animalis* subsp *lactis*	Incidence of eczema	10^9^ CFU	In the human trials reduced the incidence of eczema	[43]
*B. longum* BB536 *B. infantis* M-63, *B. breve* M-16V	Allergic rhinitis	3 × 10^9^, 1 × 10^9^ and 1 × 10^9^ CFU	In a human trial, improved seasonal allergic rhinitis symptoms and quality of life in children with pollen-induced allergic rhinitis and intermittent asthma	[44]

**Table 3:** Common probiotics used in oncology conditions.

Probiotic	Condition	Concentration	Evidence	Ref
Mixture of six viable strains *L. acidophilus* BCMC^®^ 12, *L. lactis* BCMC^®^ 12, *L. casei subsp* BCMC^®^ 12, *B. longum* BCMC^®^ 02120, *B. bifidum* BCMC^®^ 02,290, and *B. infantis* BCMC^®^ 02129	Post-surgical colorectal cancer	30 × 10^10^ CFU	Prevented post-surgical complication and decreased the levels of pro-inflammatory cytokines (TNF-α, IL-17A, IL-17C, IL-22, IL-10, and IL-12) in colorectal cancer patients	[63]
*L. acidophilus* BCMCR 12130, *L. casei* BCMCR 12313, *L. lactis* BCMCR 12451, *B. bifidum* BCMCR 02290, *B. longum* BCMCR 02,120, and *B. infantis* BCMCR 02129	Colorectal cancer	30 × 10^10^ CFU	In colorectal cancer patients improved the quality of life, decreased the levels of inflammatory biomarkers and mitigated the negative effects of chemotherapy	[64]
*L. acidophilus* (BCMCTM12130) *L. casei* (BCMCTM12313) *L. lactis* (BCMCTM12451) *B. bifidum* (BCMCTM02290) *B. longum* (BCMCTM02120) *B. infantis* (BCMCTM02129)	Colorectal cancer surgery	30 × 10^10^ CFU	Recovered the normal gut function in patients after colorectal cancer surgery and reduced duration of hospital stay	[65]
*B. infantis* *L*. *acidophilus* *E. faecalis* *B. cereus*	Gastric cancer	10^6^, 10^6^, 10^6,^ and 10^5^ CFU	Improved the immune responses, decreased inflammation, and restored the microbial diversity in gastric cancer patients subjected to gastrectomy	[66]
*B. lactis* Bl-04 (ATCC SD5219) *L. acidophilus* NCFM (ATCC 700396)	Colon cancer	1.4 × 10^10^ and 7 × 10^9^ CFU	Increased the abundance of beneficial butyrate-producing bacteria in the mucosa, tumors, and fecal samples of patients with colon cancer	[67]

**Table 4:** Probiotics associated with the management of neurological disorders.

Probiotics	Condition	Concentration	Evidence	Ref
*B. coagulans* MTCC 5856	Depressive disorder in IBS patients	2 × 10^9^ CFU	The human trials adjusted the depression score and reduced serum myeloperoxidase in patients with irritable bowel syndrome	[79]
*L. helveticus* R0052 and *B. longum* R0175	Major depressive disorder	10 × 10^9^ CFU	Improved the BDI score in patients with depressive disorder through the regulation of the kynurenine to tryptophan ratio	[80]
*L. helveticus R0052 and B. longum R0175*		3 × 10^9^ CFU	Daily administration significantly improved overall mood, anhedonia, decreased anxiety, and ameliorated quality of sleep in humans	[81]
*B. bifidum* W23, *B. lactis* W51, *B. lactis* W52, *L. brevis* W63, *L. casei* W56, *L. lactis* W58, *L. salivarius* W24, *L. acidophilus* W37, and *L. lactis* W19	Depression	1 × 10^10^ CFU	Decreased cognitive reactivity in human trials, a variable associated with susceptibility to depression	[82]
	Migraine	2.5 × 10^9^ CFU	Had a positive impact on the frequency and severity of migraine in human trials	[83]
*L. plantarum P8*	Stress/anxiety	2 × 10^10^ CFU	Improved stress and anxiety symptoms through the ability to enrich the diversity of neurotransmitter-synthesizing/consuming species-level genome bins and microbial neuroactive metabolites	[84]

**Table 5:** Probiotics speculated to exhibit anti-Alzheimer activity.

Probiotics	Condition	Concentration	Evidence	Ref
*B. breve* A1	Patients with mild cognitive impairment	2.0 × 10^10^ CFU	Can maintain cognitive function presented to significantly lift MMSE and improve POMS2 and GSRS scores	[127]
*L. acidophilus*, L. casei, B. bifidum, and *L. fermentum*	AD Patients	2 × 10^9^ CFU	Improved MMSE and significantly decreased MDA, hs-CRP levels, and HOMA-B index	[128]
*L. casei* W56, *L. lactis* W19, *L. acidophilus*, W22, *B. lactis* W52, *L. paracasei* W20, *L. plantarum* W62, *B. lactis* W51, *B. bifidum* W23, and *L. salivarius* W24		Not specified	Influenced gut bacteria composition and serum tryptophan metabolism	[112]
*B. bifidum*, *B. longum*, and selenium		2 × 10^9^ CFU + 200 μg	Improved the features of metabolic profile and refined cognitive functions and significantly increased TAC and GSH, regulated TNF-α, PPAR-γ, and LDLR	[129]
*Acetobacter aceti*, *L. delbrueckii*, *L. fermentum*, *L. fructivorans*, *E. faecium*, *L. kefiranofaciens*		7.5 × 10^7^ CFU	Induced beneficially preventive effects on cognitive dysfunction, systemic inflammation, oxidative stress, and cell damage, etc.	[130]
*B. longum*, *L. acidophilus*, and *E. faecalis*	Elderly patients for elective orthopedic/colorectal surgery	<1.0 × 10^7^ CFU	Prevented the postoperative cognitive impairments	[131]

## Data Availability

Not applicable.
